# Comprehensive experimental investigation of the effective parameters on stability of silica nanoparticles during low salinity water flooding with minimum scale deposition into sandstone reservoirs

**DOI:** 10.1038/s41598-022-20595-9

**Published:** 2022-10-01

**Authors:** Masoud Bijani, Ehsan Khamehchi, Mehdi Shabani

**Affiliations:** Department of Petroleum Engineering, Amirkabir University of Technology (Tehran Polytechnic), Tehran, Iran

**Keywords:** Nanoscience and technology, Fossil fuels, Engineering, Chemical engineering

## Abstract

Recent studies showed the high potential of nanofluids as an enhanced oil recovery (EOR) agent in oil reservoirs. This study aimed to investigate the effects of salts and ions, the salinity of aqueous solution, total dissolved solids (TDS), scale deposition of mixing brines, surface charge as zeta potential (ZP) value, and pH of injected brines as low salinity water (LSW) on the stability of silica nanoparticles (NPs). The experiments were conducted on the stability of silica NPs at different concentrations and brines to determine optimum salinity, dilution, cations, and anions concentrations. The results showed that 10 times diluted seawater (SW#10D) was optimum low salinity water (OLSW) as injected LSW and water-based nanofluids. Results showed that by decreasing the salinity, increasing seawater dilution, and removing Mg^2+^ and Ca^2+^ cations, the amount of scale deposition decreased, and the brine's brine's brine stability of NPs in brine improved. At the optimum salinity and dilution conditions, compared with other salinities, there was less scale formation with more nanofluid stability. Obtained results from ZP measurements and dynamic light scattering (DLS) showed that by removing divalent ions (Mg^2+^ and Ca^2+^) of water-based nanofluid (low salinity hard water (LSHW) composition), more NPs were attached to the surface due to the reduction in repulsive forces between the NPs. Therefore, at optimum low salinity soft water (OLSSW), more wettability alteration occurred compared with optimum low salinity hard water (OLSHW) due to the more stability of NPs in OLSSW. The obtained results from the contact angle measurements, surface adsorption of the NPs by FESEM images, and ZP measurements showed that the predominant mechanism in enhancing oil recovery by nanofluid was the wettability alteration by disjoining pressure. According to wettability alteration results, the silica NPs with an optimized concentration in the optimized LSHW and LSSW compositions could be improved the wettability alteration by up to 23.37% and 55.81% compared with the without NPs. The optimized LSSW compared with LSHW composition could be improved the wettability alteration by up to 11.69%. In addition, OLSSW-based nanofluid compared with OLSHW could be increased wettability alteration toward strongly water-wet by up to 33.44%.

## Introduction

In recent years, due to growth in the energy demand, it is remarkably important to improve oil recovery obtains before abanding currently oil wells to the newly discovered ones. Numerous enhanced oil recovery (EOR) technologies have increased in the past decades to improve the oil recovery of remaining oil trapped in the oil reservoir^1^. The chemistry of the injected water in the form of salinity as low salinity water (LSW) or modified ionic composition in the form of smart water (SW) has an effective impact on the EOR^2–4^. In the LSW injection method, by changing the composition and concentration of ions (cations and anions) present in the water, the injected fluid is shifted to high compatibility with the rock and reservoir fluids^5–7^. The dominant mechanisms of LSW flooding are reservoir rock wettability alteration ^8,9^, fine migration ^10,11^, interfacial tension (IFT) reduction ^12^, multi-ion exchange ^13–15^, and double-layer expansion ^16^. However, the main mechanism is addressed as wettability alteration in most publications ^17^.

One of the aspects of nanotechnology is applying the new chemical enhanced oil recovery (C-EOR) in oil reservoirs. The application of nanoparticles (NPs) rather than other chemical materials shows this method's efficiency and economic advantages. The NPs can be applied in nanofluid, and LSW flooding operations contain NPs, ions, and optimum composition as EOR agents in the injection water (IW) ^18^. NPs, as nanofluids, are proposed as alternative flooding water-based EOR (i.e., LSW or SW) due to their specific characteristics, small size, high surface area, high surface-to-volume ratio, free movability, and dispersion ability in porous media ^19–21^. Numerous studies have been done on the application of NPs in EOR. Hendraningrat et al.^22^ studied the silica NPs possibility to improve oil recovery in sandstone rocks and examined effective concentration. They concluded that higher concentrations of NPs had more formation damage in the rocks. However, increasing NP_S_ concentration illustrated a change in wettability, and their results have shown that more recovery is not assured. Bazazi et al.^23^ investigated the influence of silica NPs on wettability alteration. They conducted a series of experiments on the impact of silica nanofluids on EOR. They observed that the efficiency of NPs obtained more oil recovery factors than water flooding and chemical flooding methods. Yuyang et al.^24^ studied the impact of modified silica NPs on wettability alteration of oil-wet cores. They used the UV-spectroscopy and dynamic light scattering (DLS) methods to determine NPs stability, contact angle, and imbibition tests to investigate the efficiency NPs in improving EOR. The results showed that modified silica NPs could enhance wettability alteration from oil-wet to water-wet. Olayiwola and Dejam ^25^ studied the efficiency of silica NPs on the wettability alteration during LSW and surfactant flooding in carbonate reservoirs. They observed that injection of NPs after LSW could alter wettability from oil-wet to water-wet. They examined important mechanisms for improving EOR performance: wettability alteration, reduction of IFT, and viscosity modification. They reported that using NPs during the LSW was more efficient and economical for improved EOR mechanisms in carbonate reservoirs. Rafiei and Khamehchi^26^ presented similar outcomes in artificial sandstone core samples. Mahmoudpour et al.^9^ investigated silica NPs' effect and SW on oil recovery. Their results showed that the dispersed silica NPs in SW could remarkably improve the wettability alteration mechanism; also, it was the best influence on oil recovery by raising the liquid viscosity. Ezzati and Khamehchi^27^ studied the stability of silica NPs in low salinity brine and investigated the ZP (zeta potential ) effect on stability at different salts. They concluded that nanofluids without Mg^2+^ and Ca^2+^ ions had the highest stability. Songolzadeh and Moghadasi^28^ investigated stabilizing silica NPs in saline environments for wettability alteration of oil-wet reservoir rocks. They used two surfactants Cetyl Trimethyl Ammonium Bromide (CTAB) and Sodium Dodecyl Sulfate (SDS), for stabilizing NPs in high saline waters. Their results showed that nanofluid with 0.05 wt.% silica NPs and SDS was the highest efficiency on wettability alteration. In addition, silica NPs have a remarkable impact on oil/brine contact angle; as a result, NPs can be applied to improve oil production by changing the wettability. Al-Anssari et al.^29^ studied the impact of salinity on silica NPs stability in saline environments. Their results showed that increasing salt NaCl concentration had a considerable impact on the ZP value of nanofluids.

Unlike the previous experimental works ^1,9,18,25,27,30–36^, this study has tried to extend the investigation of the effective parameters on the stability of silica NPs at different LSW compositions. These parameters included the effect of salinity, times diluted seawater, formation damage as scale deposition, type and concentration of ions and salts, pH, surface charge, and NPs concentration in aqueous solution.

This paper has the following novel points: (1) unlike the previous experimental works, the paper provides physiochemical insights comprehensively (effects of ions, pH, surface charges, salinity, dilution ratio) to examine nanofluid utilization to improve wettability alteration and enhance oil recovery; (2) investigation of essential and effective factors on stability and compatibility of dispersed NPs in optimum low salinity hard water (OLSHW) and optimum low salinity soft water (OLSSW) with minimum scale deposition during LSW flooding is confirmed as an effective method to improve wettability alteration in sandstone oil reservoirs.

**Figure 1:**
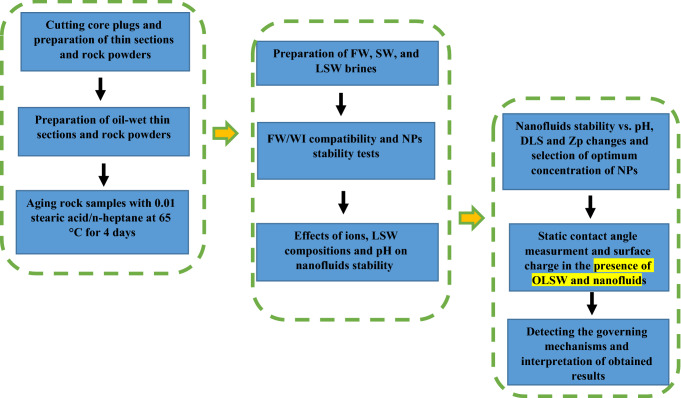
Schematic diagram of the workflow.

This study investigates the potential of silica NPs at different concentrations as the new agent and wettability modifier in a sandstone reservoir through contact-angle, Field Emission Scanning Electron Microscopy (FESEM), ZP, DLS, pH, and brine compatibility measurements. Experimental tests are conducted with and without NPs to study the compatibility and scaling potential, stability of NPs in LSW brines, and wettability alteration of different LSW compositions before and after dispersing with NPs. In addition to examining different LSW compositions associated with NPs considering wettability alteration, scale-risk, and surface charge perspectives. Experimental investigations into the rock/fluid (nanofluid and brine) and nanofluid/brine interactions are also conducted through the contact angel test, FESEM, and nanofluid stability behavior by compatibility test, ZP, and DLS measurements. A schematic diagram of the workflow of this study is shown in Fig. 1. This work provides an opportunity to study the possible changes in the rock and fluid characteristics (i.e., wettability) after NPs treatment during LSW flooding. In summary, this research indicates the consequence of a comprehensive study concerning the developed silica nanofluids with an optimum concentration as new agents based on LSW compositions (OLSHW and OLSSW) could be more efficient and economical for improved wettability alteration and reservoir damage in minimum scale formation.

## Materials and methods

### Materials

#### Brines

The brine samples were made in the laboratory by dissolving specified quantities of different salts, salts at high purity from Merck Chemicals (purity of 99.5%). The salts included CaCl_2_.2 H_2_O, NaCl, KCL, Na_2_SO_4_, NaHCO_3_, and MgCl.6H_2_O dispersed in deionized water. The brine compositions of the IW (seawater) and formation water (FW) as a candidate for reservoir water are tabulated in Table 1. The FW composition tabulated in Table 1 was the water composition of one of the sandstone oil reservoirs in Southwest Iran. The LSW solutions were prepared based on the initial Persian Gulf seawater composition (Table 2).

**Table 1:** Complete water compositions of the FW and seawater**.**

Ions	Unit	Formation water	Persian Gulf seawater
Na^+^	mg/l	59,142.47	12,653
K^+^	mg/l	0	420
Ca^2+^	mg/l	13,500	498
Mg^2+^	mg/l	1725	1408
SO_4_^2-^	mg/l	449	3037
Cl^-^	mg/l	120,444.44	22,598
HCO_3_^-^	mg/l	293.68	73
TDS	mg/l	195,671.04	40,687
Density	g/ml	1.126	1.026
Total Alkalinity	mg/l as HCO_3_	293.06	73
Salinity	mg/l as NaCl	109,732.07	37,173.71
Density	gr/cc	1.06	1.026
pH	–	6.5	8.138
Ionic strength	Molal	1.73	0.829
EC	ms/cm at 25 °C	130	58

**Table 2:** Composition of diluted waters with different compositions of the Persian Gulf as IW.

Parameter	Unit	Seawater	SW#2D	SW#5D	SW#10D
Na^+^	mg/l	12,653	6326.5	2530.6	1265.3
K^+^	mg/l	420	210	84	42
Ca^2+^	mg/l	498	249	99.6	49.8
Mg^2+^	mg/l	1408	704	281.6	140.8
SO_4_^2-^	mg/l	3037	1518.5	607.40	303.7
Cl^-^	mg/l	22,598	11,299	4519.6	2259.8
HCO_3_^-^	mg/l	73	36.5	14.6	7.3
TDS	mg/l	40,687	20,343.5	8137.4	4068.7
Density	gr/cc	1.026	1.012	1.003	1
pH	–	8.138	8.138	8.138	8.131

#### Nanoparticles

In this study, types of metal oxide NPs, including aluminum (Al_2_O_3_), magnesium (MgO), zinc (ZnO), copper (CuO) oxides, and silicon dioxide (SiO_2_) were used. The NPs used in this work were purchased from the US Research Nanomaterial Inc, Houston, TX, USA. The characteristics of the metal oxide NPs are listed in Table 3.

**Table 3:** The characteristics of metal oxide NPs used in this study.

Nanoparticles	Specific area (m^2^/g)	Average diameter (nm)	Morphology	Purity (%)	Appearance
MgO	> 60	20	Tetrahedron	> 98	White powder
ZnO	20–60	10–30	Nearly spherical	99	Cream
Al_2_O_3_	90—160	10–20	Spherical	99.99	White powder
CuO	20	25–55	Nearly spherical	99	Black powder
SiO_2_	180–600	20–30	Spherical	99.99	White powder

#### Sandstone rock samples

Thin sections of specimens at a 3.6 cm diameter from sandstone rock were provided for contact angle tests. FESEM images of surface rock were taken to investigate surface adsorption of NPs and surface changes due to the effect NPs and LSW. Also, the rock powder was prepared from the sandstone rock sample for investigation of surface charge and ZP tests. The rock powder was prepared in the same procedure with thin slices aged with a strong fatty acid (0.1 molars of stearic acid) and normal heptane at a temperature reservoir in the oven for four days until rock powders were obtained from oil-wet. An x-ray fluorescence spectrometry (XRF) analysis was conducted to evaluate the core's rock composition because rock lithology could directly affect surface charge analysis. Table 4 shows the XRF analysis results for determining rock mineralogy.

**Table 4:** The results of XRF analysis of core samples (%).

Element	SiO_2_	Al_2_O_3_	BaO	CaO	Fe_2_O_3_	K_2_O	MgO	MnO	Na_2_O	P_2_O_5_	SO_3_	TIO_2_	LOI
Value (%)	36.59	2.14	<	29.54	1.65	0.45	2.22	>	0.33	0.08	0.09	0.13	>

#### Crude oil

In this study, crude oil from one of the southwestern Iranian reservoirs was used in the experiments. Tables 5 and 6 show the composition and physical properties of the crude oil and chemical composition used in this study. Also, API (American Petroleum Institute Gravity) indicates the gravity or density of crude oil and liquid petroleum products.

**Table 5:** Composition of utilized crude oil in the experiments**.**

Components	C_1_	C_2_	C_3_	iC_4_	nC_4_	iC_5_	nC_5_	C_6_	C_7_	C_8_	C_9_	C_10_	C_11_	C_12_^+^	H_2_S	CO_2_
Reservoir oil (mol %)	45.59	7.02	4.28	0.87	2.13	0.88	0.88	4.78	1.33	3.14	1.72	2.04	1.78	23.21	0	0.21

**Table 6:** The physical properties of crude oil in the experiments.

M.W (g/mol)	Density (g/cm^3^)	API	Viscosity (cP)
86	0.64	32.24	0.41

## Methods

### Preparation of nanofluids

The observational investigation performed the experiments on nanofluids' stability at different LSW and times diluted seawater (SW#XD) compositions at reservoir temperature (65 °C). In this study, for the preparation of different nanofluids, five different concentrations of SiO_2_ NPs in the ranges of 500, 1000, 2000, and 2500 ppm were first added to 1000 ml of the seawater and LSW compositions [without, 2, 5, and 10 times diluted seawater (SW#0D, SW#2D, SW#5D, and SW#10D)] and mixed by magnetic stirrers for 30 min. Then, the solutions were endured for 1 h of ultrasonic radiation using the Ultrasonic Probe DA UP-400 (Development of Ultrasonic Technology Co., Iran) apparatus. The ultrasonic probe apparatus with breaking nanoparticle clusters could improve nanofluids stability and inhibit NPs agglomeration and deposition in the base fluid^30^.

### Determination of scale deposition

An experimental test for evaluating the compatibility of FW with injected brines was applied to the evolution of the amount of scale deposition and the determination of optimum low salinity water (OLSW). This test was conducted at a reservoir temperature (65 °C). First, the Persian Gulf was considered a candidate for IW. Also, LSW samples with different dilution times were prepared according to this composition, including SW#0D, SW#2D, SW#5D, and SW#10D brines. All synthetic brines included FW and injection waters made in 1-L volume for experiments. After, these samples were mixed with formation brine at a mixing proportion of 1:1 or a mixing ratio of 50% IW/FW. 100 cc of each brine was filtered, poured into a glass bottle, and mixed with formation brine at a 0.5 mixing proportion for each compatibility test.

Moreover, every sample was put in an oven at 65 °C for three days. Reaching the different equilibrium conditions can be associated with scale formation. Scale formation occurred due to the supersaturation of one or more types of salt in a liquid^37^. After 3 days, mixed brine samples were filtered by 0.22 µm filter paper, and then the mass of the scale was measured. As the main purpose was to investigate the mineral scale weight, a membrane filtration apparatus was used to filter the samples. Furthermore, the filters were cleaned with deionized water, and the mineral scale was free of sodium chloride (NaCl) to ensure measures of exact filter weighting. After the filters were dried in an oven at 65 °C to evaporate associated with water, only the precipitated scales remained on the filters. The laboratory scale was utilized to weigh filters' accurate mass and measure the total suspended solids (TSS) in mixed brine samples.

### Zeta potential and dynamic light scattering apparatuses

ZP is the main parameter in the NPs distribution for the nano-suspensions^38^. When the ZP has sufficient high values (without regard to the negative or positive charges), it can make sure the stability of the NPs in an aqueous solution. In contrast, lower ZP values cause NPs agglomeration in aqueous solution^38,39^.

In this study, the ZP, the hydrodynamic diameter, and the dispersion stability of NPs by DLS (Malvern NanoSizer ZS) were examined. In addition, the surface charge of solutions was estimated by the Malvern Zeta-sizer Nano ZS instrument concerning sandstone grains dispersed in the wanted brine. The surface charge value was listed in the ZP value of various samples. The dispersed sandstone grains in brines were provided by combining 0.5 g of milled sandstone particles with 50 ml of brine, representing 1% weight of the liquid suspension. The solutions were sonicated for 30 min with a sonication tool and allowed to hold for 2 days to give the equilibration condition. An adequate value of ZP was elected about the average of three analyses for each sample.

### Aging procedure

**Figure 2:**
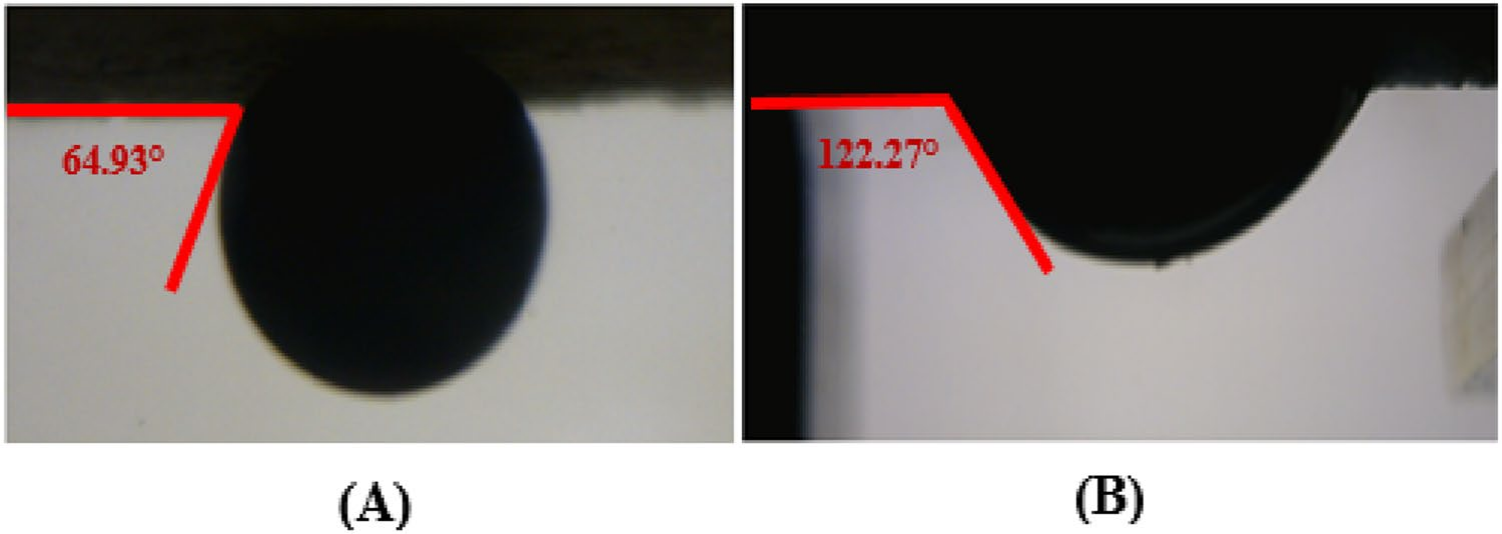
Images of contact angle changes of an oil droplet on the sandstone surface **(A)** before and (**B**) after wettability alteration with stearic acid and n-heptane.

As the surface of sandstone rocks was generally water wet due to chemical structure, a stearic acid/n-heptane mixture was used, and the primary wettability of rocks was changed toward oil-wet. Many researchers related to LSW and other EOR mechanisms reported that fatty acids could alter the rock wettability toward oil-wetness under these conditions^40–44^. Utilizing this method could extensively decrease the time needed for aging the samples. The sandstone rock samples were oil wetted by 0.01 molarity of stearic acid/*n*-heptane liquid at 65 °C for 4 days (Fig. 2).

### Contact angle apparatus

**Figure 3:**
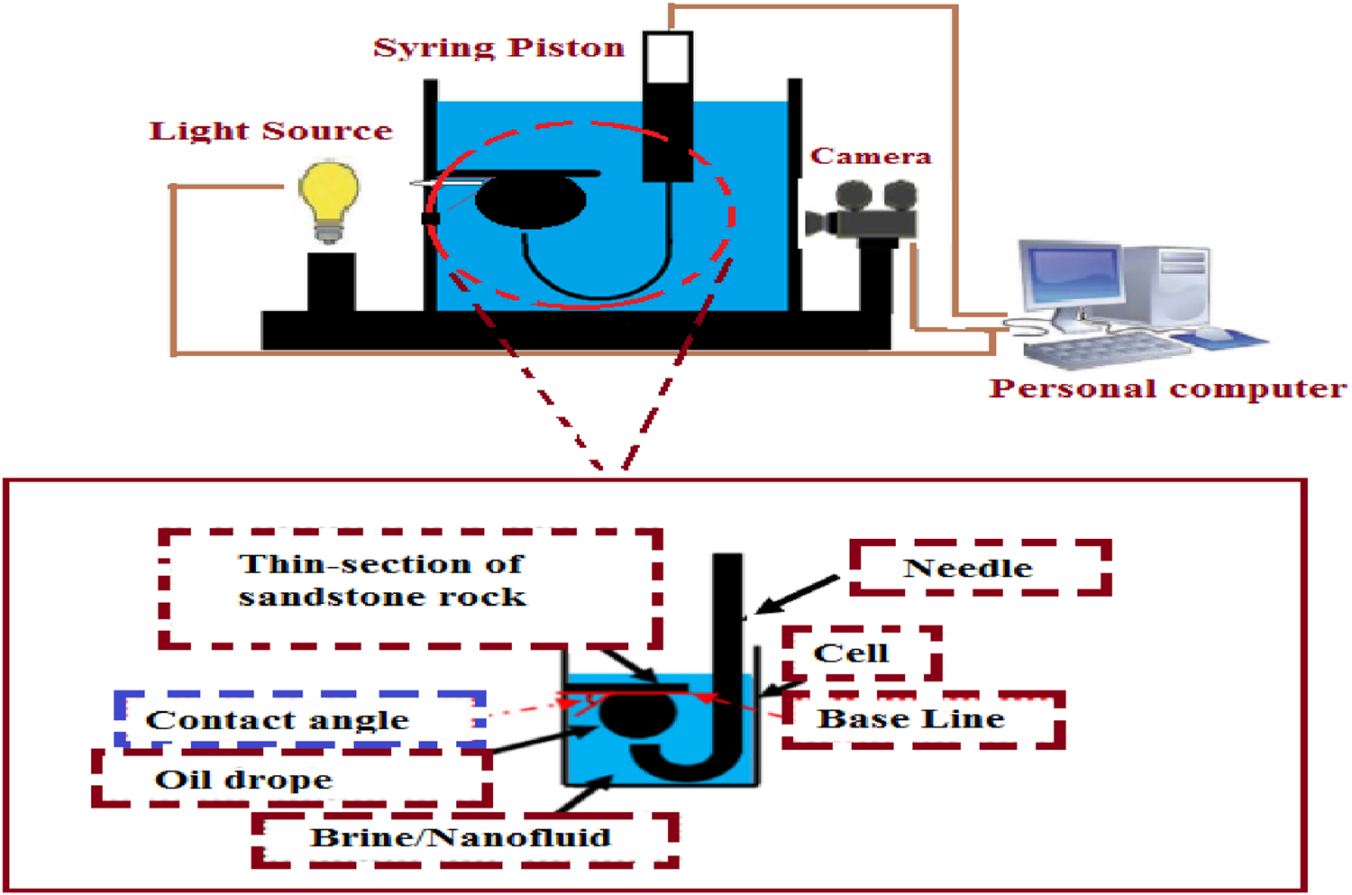
Schematic drop shape analyzer (DSA) diagram for contact angle measurement setup.

A drop-shape analysis (DSA) apparatus was utilized to measure the equilibrium oil contact angle/aqueous solution on the sandstone rock surface at the reservoir temperature and ambient pressure conditions. Contact angle values of thin section specimens before and after nanofluids injection at different NPs concentrations and LSW compositions were estimated at reservoir temperature. Figure 3 shows the schematic diagram of the contact angle apparatuses.

### pH effects

To examine the effect of pH values on the surface charge and stability of NPs in LSW brines, the pH values of aqueous solutions were measured with a pH lab. The average particle size and ZP values in brines with and without the rock powders were measured immediately. While these measurements were in progress, the pH of solutions was changed and modified by adding diluted HCl or NaOH solutions. The LSW brines with and without NPs were dispersed in rock powders on magnetic stirring and after sonicated in an ultrasonic apparatus for 30 min at room temperature.

## Results and discussion

### Evaluation of the stability of NPs at different brines

The stability of NPs in electrolyte solutions is an essential parameter in the performance evaluation of nanofluids. Experiments on nanofluids' stability in various injected waters were performed by observation under reservoir temperature (65 °C). According to this approach, each NPs was weighed at the relevant weights and added to the brines. After ultra-sonication of the solutions, they were kept in the oven at reservoir temperature for 7 days. Table 7 shows metal oxide NPs of aluminum dioxide (Al_2_O_3_), magnesium oxide (MgO), zinc oxide (ZnO), and copper oxide (CuO) that all NPs settled in tubes in different injected waters with different salinities and dilution times seawater after ultra-sonication. Eventually, only silicon dioxide (SiO_2_) NPs were stabilized in SW#10D, and silica NPs did not stabilize in SW#0D, SW#2D, and SW#5D brines. As shown in Table 7, the silica nanofluids with concentrations of 500, 1000, and 2000 ppm were stabilized in SW#10D. Thus, these concentrations of NPs were the saturation concentration in which the optimal ZP and DLS values were obtained for the silica nanofluids at the temperature reservoir (65 °C).

**Table 7:** Observation investigation of the stability of NPs at different salinities and dilution ratio of seawater as IW for 7 days.

Nanoparticle	The concentration of NPs in brines (ppm)				NPs stability in brines
	500	1000	2000	2500	
SiO_2_	Stable	Stable	Stable	Unstable	Only stable at SW#10D
CuO	Unstable	Unstable	Unstable	Unstable	Unstable
ZnO	Unstable	Unstable	Unstable	Unstable	Unstable
Al_2_O_3_	Unstable	Unstable	Unstable	Unstable	Unstable
MgO	Unstable	Unstable	Unstable	Unstable	Unstable

### Effect of scale formation of brines on nanofluid stability

One of the main problems during water injection projects is formation damage by inorganic scale formation due to mixing the incompatible injected water with FW at reservoir conditions ^37,45,46^. The potential of mineral scale deposits due to the incompatibility of injected water and the FW during the injection of LSW and SW flooding has been less considered ^6^.

The obtained results from the compatibility tests due to mixing FW and injection waters in SW#0D, SW#2D, SW#5D, and SW#10D compositions with a mixing ratio 1:1 (% 50 to % 50 WI/FW) of one of the oil fields in southwestern Iran indicated that the minimum scale formation happened in SW#10D as OLSW composition. These results correspond to the effects of salinity and dilution times on the stability of NPs in different brines. Table 8 presents the quantity and type of scale formation obtained from mixing brines. Moreover, the OLI ScaleChem software could predict scale precipitation's amount and species distribution due to mixing various brine compositions under water and CO_2_ flooding operations ^46–48^. As a result, this software was used to determine the minimum scale deposition that could occur during the LSW samples for scale-up to oil reservoirs. It is noticed that the prediction and control of scale deposition are essential problems in LSW flooding operations ^2,47^. The software can calculate saturation ratio (SR) and saturation index (SI) terms to predict scale formation for an aqueous solution. SR and SI are defined as follows ^37,46^ :

1 $$ SR = \frac{{\alpha_{Me} .\alpha_{An} }}{{K_{sp(P,T,I)} }} = \alpha_{Me} .\alpha_{An} - K_{sp(P,T,I)} $$

2 $$ SI = \log (SR) = \log_{10} \{ \frac{{\alpha_{Me} .\alpha_{An} }}{{K_{sp(P,T,I)} }}\} = \log \{ \alpha_{Me} .\alpha_{An} \} - \log K_{sp(P,T,I)} $$

**Table 8:** The scale mass deposited for each LSW composition at different times diluted seawater.

Types of brine	The total mass of scale formation (mg/L)	SR	SI	The total mass of scale formation determined by OLI ScaleChem software (mg/L)
SW#0D + FW	153	SR (CaCO_3_) = 7.37	0.87	49.07
		SR (CaSO_4_) = 1.23	0.089	394.18
SW#2D + FW	125	6.6	0.819	43.89
SW#5D + FW	121	6.15	0.788	40.7
SW#10D + FW	60	6.12	0.786	39.7

The activity of the ion species**,**${\mathrm{\alpha }}_{i}$, is also defined as follows:

3 $$ \alpha_{i} = [C_{i} ]\gamma_{i} $$

where Ci is the ionic concentration of each ion and ${\upgamma }_{\mathrm{i}}$ is the activity coefficient of the i species, respectively.

The aqueous solution is supersaturated, and the scale tends to form when the SR and SI are greater than 1.0 and 0, respectively. In contrast, the aqueous solution is unsaturated, and mineral scales cannot be formed when the SR and SI are lower than 1.0 and 0, respectively ^37^. As shown in Table 8, SW#10D had the minimum quantity of mineral scale with the FW, so SW#0D, SW#2D, and SW#5D created higher scale deposition. Therefore, the simulation results were highly compatible with the experimental. These results proved that the SW#10D with minimum scale deposition had the highest stability of NPs of the other LSW brines. A direct relationship was observed between scale formation and stability of NPs in LSW. Consequently, scale deposition negatively affected the stability of NPs in brine. As a result, NPs with optimum concentration could reduce scale formation in solution^49–51^. Also, scale deposition is inversely related to the stability of NPs in brine ^49^.

### Effect of salinity, TDS, and dilution times on nanofluid stability

The NPs concentrations in base fluids can change after injection into oil reservoirs because of the deposition and adsorption of NPs on the rock surface ^52^. As shown in Tables 7 and 9, the NPs stability was increased with decreasing salinity ^53–56^. Therefore, it is recognized that the high salinity of solution has a screening impact on the electrostatic repulsion forces between NPs in solution, leading to an accelerated coalescence and deposition of NPs. As can be seen in Table 7, the stability of silica NPs in SW#10D was the highest value of other times diluted seawater compositions. Also, as shown in Table 9, the salinity, TDS, and ionic strength in SW#10D had the lowest values compared with other compositions, and only NPs were stabilized in SW#10D composition (Table 7). As a result, the salinity and TDS were effective parameters on NPs stability in the solution. Increasing salinity and TDS could reduce the NPs stability in the injection brines due to the rising ionic strength; therefore, it could decrease the ZP value and raise the average particle size value of nanofluids^54^. Also, Tables 7, 8 and 9 show a direct relation between salinity, electrical conductivity (EC), and TDS of brines and Mineral scale deposition with nanofluid stability.

**Table 9:** TDS, salinity, EC, and IS values of mixing diluted waters with different compositions of the Persian Gulf as IW with FW.

Parameter	Unit	Seawater	SW#2D	SW#5D	SW#10D
TDS	mg/l	40,687	20,343.5	8137.4	4068.7
Salinity	mg/l as NaCl	37,173.71	18,586.855	7434.74	3717.37
EC	mS/cm at 25 °C	58	29	11.6	5.8
Ionic strength	Molal	1.51	1.13	1.19	1.15

### Effect of types of ion and compositions of brine on nanofluid stability

**Figure 4:**
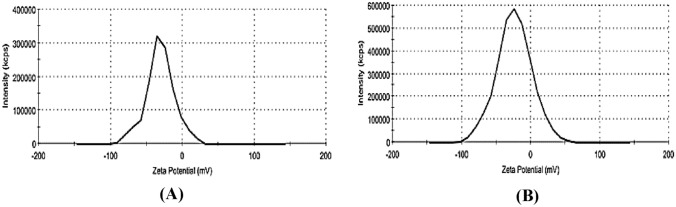
(**A**) More stability of NPs in LSSW without calcium and magnesium ions (ZP = −30.8), (**B**) lower stability of NPs in LSHW with calcium and magnesium ions (ZP = −23.9).

**Figure 5:**
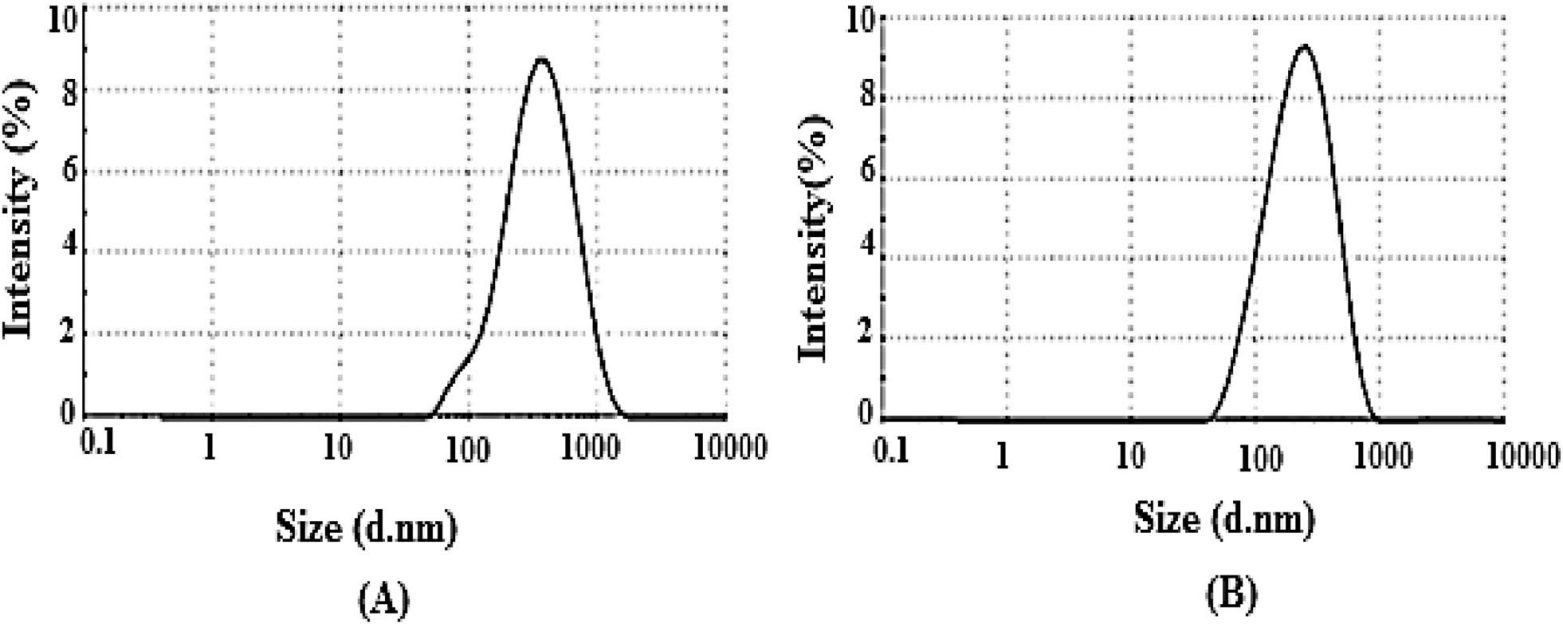
Particle size distributions of silica nanofluids by intensity (**A**) the effects of divalent cations (Ca^2+^ and Mg^2+^). (**B**) LSSW without divalent cations (Ca^2+^ and Mg^2+^).

Table 10 shows the composition of LSW with and without Ca^2+^ and Mg^2+^ ions. Results of the ZP measurements of silica NPs in LSSW and LSHW compositions are given in Table 11. The ZP values were the mean of 3 measurements for the sample. For example, Fig. 4 shows the ZP distribution curve of silica NPs for sample 1 containing LSHW and LSSW compositions. In this figure, the peak ZP values (highest values) of nanofluids from LSSW and LSHW solutions were about −30.8 mV and −23.9 mV, respectively. Also, the silica NPs size distribution curves are shown in Fig. 5. Based on this figure, the peak silica NPs size distribution curves (highest values) of nanofluids prepared based on LSSW and LSHW are diameters of about 207 nm and 227 nm, respectively. As shown in Figs. 4 and 5 and Table 11, the stability of low salinity soft water-based nanofluid (LSSWN) without Ca ^2+^ and Mg^2+^ ions was higher than low salinity hard water-based nanofluid (LSHWN). As cation valency was increased, therefore nanofluid instability was increased. Table 11 shows that active ions of Ca ^2+^ and Mg^2+^ in OLSW composition could reduce the nanofluid stability^55^. As shown in Fig. 5, this effect of ions in agreement with the DLS demonstrated that the presence of MgCl_2_ and CaCl_2_ salts compared with NaCl, KCl, Na_2_SO_4_, and NaHCO_3_ salts had a negative effect on the stability of SiO_2_ nanofluids. The mean particle size of nanofluid in the presence of CaCl_2_ and MgCl_2_ salts was about 1.5 times higher than NaCl after 7 days. Also, as can be seen in Tables 10 and 11, silica NPs in brines containing CaCl_2_ and MgCl_2_ salts than in brines containing NaCl, KCl, Na_2_SO_4_, and NaHCO_3_ salts that leads to a silica nanofluid with less stability. Also, the results showed that the type of salt could control the efficient concentration of NPs and change the size of dispersed NPs in the LSW as a carrier fluid.

**Table 10:** OLSHW and OLSSW compositions with and without Mg^2+^ and Ca^2^ ions for preparation of nanofluids.

Type of salt	Value (mg/lit)	
	OLSHW composition	OLSSW composition
MgCl_2_.6H_2_O	0.275	–
CaCl_2_.H_2_O	0.08	–
Na_2_SO_4_	0.22	0.22
NaCl	3.719	3.719
KCl	0.08	0.08
NaHCO_3_	0.01	0.01

**Table 11:** ZP values of silica NPs in LSHW and LSSW compositions.

Composition	Sample 1	Sample 2	Sample 3	Average	Std	Uncertainty value (mV)
LSHW	−23.9	−23.5	−24.4	−23.9	0.37	−23.9 ± 0.72
LSSW	−30.8	−29.6	−29.7	−29.03	0.54	−30.03 ± 1.07

### The effects of pH and concentration of silica NPs on nanofluids stability in OLSW

**Figure 6:**
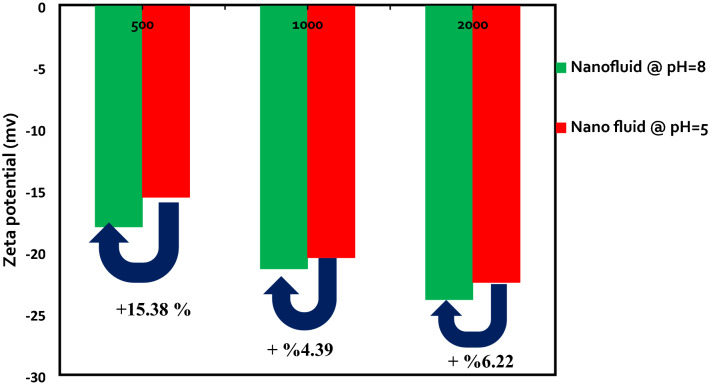
ZP vs. NPs concentration with various pH.

**Figure 7:**
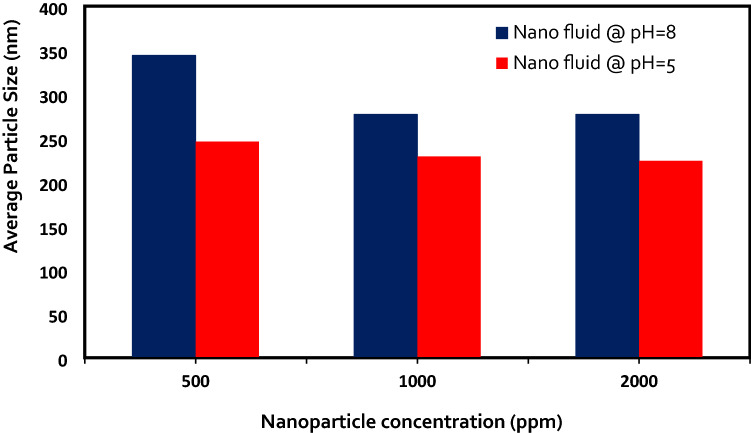
Average particle size vs. NPs concentration with various pH.

The ZP and DLS values of LSHW/LSHW-based nanofluids were measured at different pH to evaluate the effects of pH and silica NPs concentrations on the stability of silica nanofluids. Nanofluids included silica NPs in different concentrations of 500, 750, 1000, and 2000 ppm. For each solution, there is a pH in which the nanoparticle's ZP value is zero and called the Isoelectric point (IEP) ^53^. At this point, the net electric charge on the particles' surface is zero since the electrostatic repulsion force between the particles is insufficient; as a result, the sediment and lumps of NPs ^57^. If the pH of the nanofluid solution is higher than IEP, then the NPs in the solution can have a negative electrical potential. According to the literature, the IEP of the silica nanofluids is between 1.75 and 3.5 ^58^. While the pH value is higher than this value, the NPs receive a negative charge, and the ZP value increases negatively. Ionization of the reactive silanol groups (-SiOH) is the main source of the surface charge ^59^. Thus, hydroxyl groups can be adsorbed with neutral silanol groups at pH values above IEP (pH  8), and particles shift negatively charged (as shown in Fig. 6 and Table 14). Hence, at pH values below the IEP, the silanol groups react with free protonated water and create positive groups ^59^. It is well understood that ionic liquid stability is relevant to large ZP values. The NPs agglomerate happens at pH near IEP because of the consequence of low surface charge densities ^60^. Therefore, the nanofluid with a pH equal to 8 had a higher surface charge density and stability than the nanofluid with a pH equal to 5. Consequently, The results of ZP and DLS measurements in Tables 14 and 15 and Figs. 6 and 7 confirmed that the stability of the NPs remarkably altered with the pH variations.

**Figure 8:**
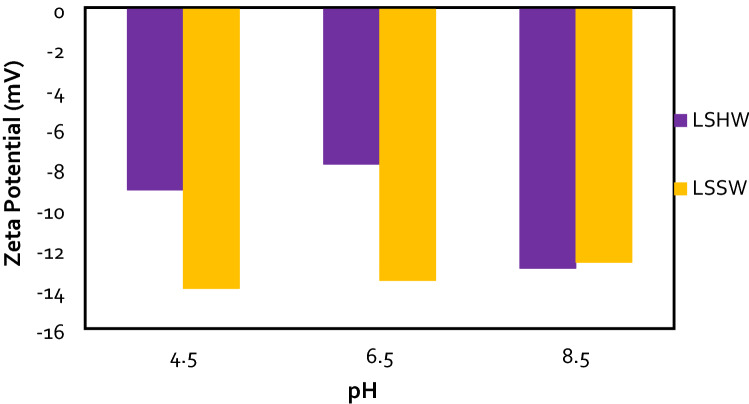
Effect of pH changes and composition of optimum diluted seawater (SW#10D) with and without divalent ions as LSSW and LSHW compositions on ZP values.

Also, screening effects of the LSHW and LSSW compositions can be seen in Tables 11, 12, 13, 14 and 15 and Figs. 6 and 7. As depicted in Tables 13 and 14, adding CaCl_2_ and MgCl_2_ salts to LSW composition as divalent cations (Ca^2+^ and Mg^2+^) could decrease the ZP values and surface charges. The reason might be due to the selective adsorption of ions on the surface of the NPs, which could prevent the particle charge. Therefore, divalent cation (Mg^2+^ and Ca^2+^) could negatively affect silica nanofluids' stability compared with OLSSW in the presence of monovalent ions (Na^+^ and HCO_3_^−^) and divalent anion of SO_4_^2−^. Moreover, as shown in Fig. 8 and Tables 13, 14, and 15, this effect of ions in agreement with the DLS results showed that MgCl_2_ and CaCl_2_ salts had a negative impact on the stability of silica nanofluid as opposed to NaCl, KCl, Na_2_SO_4_, and NaHCO_3_ salts. As can be seen in Tables 14 and 15, the DLS values of nanofluids in the presence of CaCl_2_ and MgCl_2_ salts were higher than other salts after 7 days. As mentioned above, this difference was due to lower strength of repulsive forces between silica NPs in brines containing CaCl_2_ and MgCl_2_ salts than in brines containing NaCl, KCl, Na_2_SO_4_, and NaHCO_3_ salts, led to nanofluids with less stability.

**Table 12:** Effect of types of ionic compositions on surface charge.

Uncertainty value (nm)	Std	Average particle size (nm)	Std	Average ZP (mv)	Brine
3050 ± 7.33	3.74	3050	0.89	−9.79	LSSW at pH 8
1830 ± 8.00	4.08	1830	0.82	−3.11	LSHW at pH 8

**Table 13:** ZP and DLS results of OLSSW with uncertainty in the presence of monovalent ions with various NPs concentrations at pH  8.

Concentration (ppm)	Average ZP (mv)	Std	Average particle size (nm)	Std	Uncertainty value (nm)
500 ppm SiO_2_	−24.4	1.55	316	3.27	316 ± 6.40
750 ppm SiO_2_	−19.7	1.39	198	3.27	198 ± 6.40
1000 ppm SiO_2_	−30.8	1.71	207	3.74	207 ± 7.33

**Table 14:** ZP and DLS results of OLSHW with uncertainty in the presence of divalent ions with various NPs concentrations at pH = 8.

Concentration (ppm)	Average ZP value (mv)	Std	Average particle size (nm)	Std	Uncertainty value (nm)
500 ppm SiO_2_	−18	0.82	344	2.45	344 ± 4.80
1000 ppm SiO_2_	−21.4	1.39	277	1.63	277 ± 3.20
2000 ppm SiO_2_	−23.9	1.55	277	2.16	277 ± 4.23
2500 ppm SiO_2_	Unstable	–	Unstable	–	–

**Table 15:** ZP and DLS results of OLSHW with uncertainty in the presence of divalent ions with various NPs concentrations at pH  5.

Concentration (ppm)	Average ZP value (mv)	Std	Average particle size (nm)	Std	Uncertainty value (nm)
500 ppm SiO_2_	−15.6	2.20	246	3.27	246 ± 6.40
1000 ppm SiO_2_	−20.5	1.47	229	2.45	229 ± 4.80
2000 ppm SiO_2_	−22.5	2.37	224	2.94	224 ± 5.77
2500 ppm SiO_2_	Unstable	–	Unstable	–	–

With the increasing silica NPs concentration in LSW and according to ZP measurements in Fig. 6 and Tables 13, 14 and 15, the surface charge of NPs increased and caused more force repulsion between particles, and the possibility of sticking and agglomeration of particles decreased. As a result, the average particle size in the solution decreased^61^. The results of average particle size measurements agree with ZP measurements (as seen in Figs. 6 and 7 and Tables 13, 14 and 15). Also, according to Tables 14 and 15 and Figs. 6 and 7, a critical NPs concentration (CNC) in LSW composition exists that the dispersed silica NPs in LSHW solutions become unstable at higher than this value. This CNC of silica NPs is about 2000 ppm; above this value, the surface charge of NPs reduces. Thus, this work causes less force repulsion and more attraction forces between particles, and the possibility of sticking and agglomeration of NPs increases^33^. As a result, according to the zeta and DLS values, 0.2 wt.% was the optimum concentration of silica NPs in OLSW at the temperature reservoir (149 °F).

### The effects of water composition, type of ion, pH, and NPs concentrations on rock surface charge and wettability alteration

**Figure 9:**
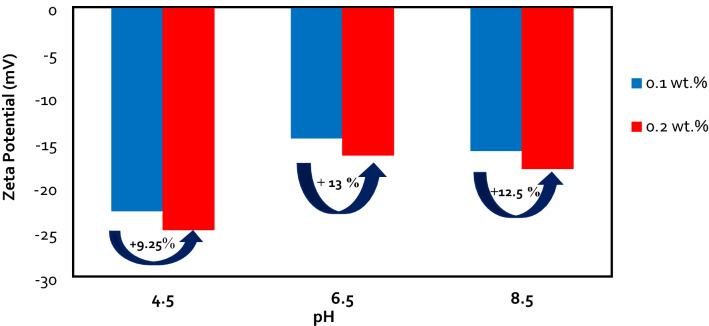
Effect of pH changes on ZP values in optimum diluted seawater (SW#10D) in the presence of silica NPs with different concentrations.

The ZP values were measured in the sandstone rock/brine (R-B) systems. The results of ZP measurements are illustrated in Figs. 8 and 9 for 1 wt.% of sandstone powder in the presence of OLSSW, OLSHW, and silica NPs. As previously mentioned, the powders of sandstone rock were aged in *n*-Heptane and 0.1 M stearic acid at 65 °C temperature and 14.7 psi pressure until rock powders became oil-wet. The ZP values shown here were the mean of 3 measurements for the sample.

**Figure 10:**
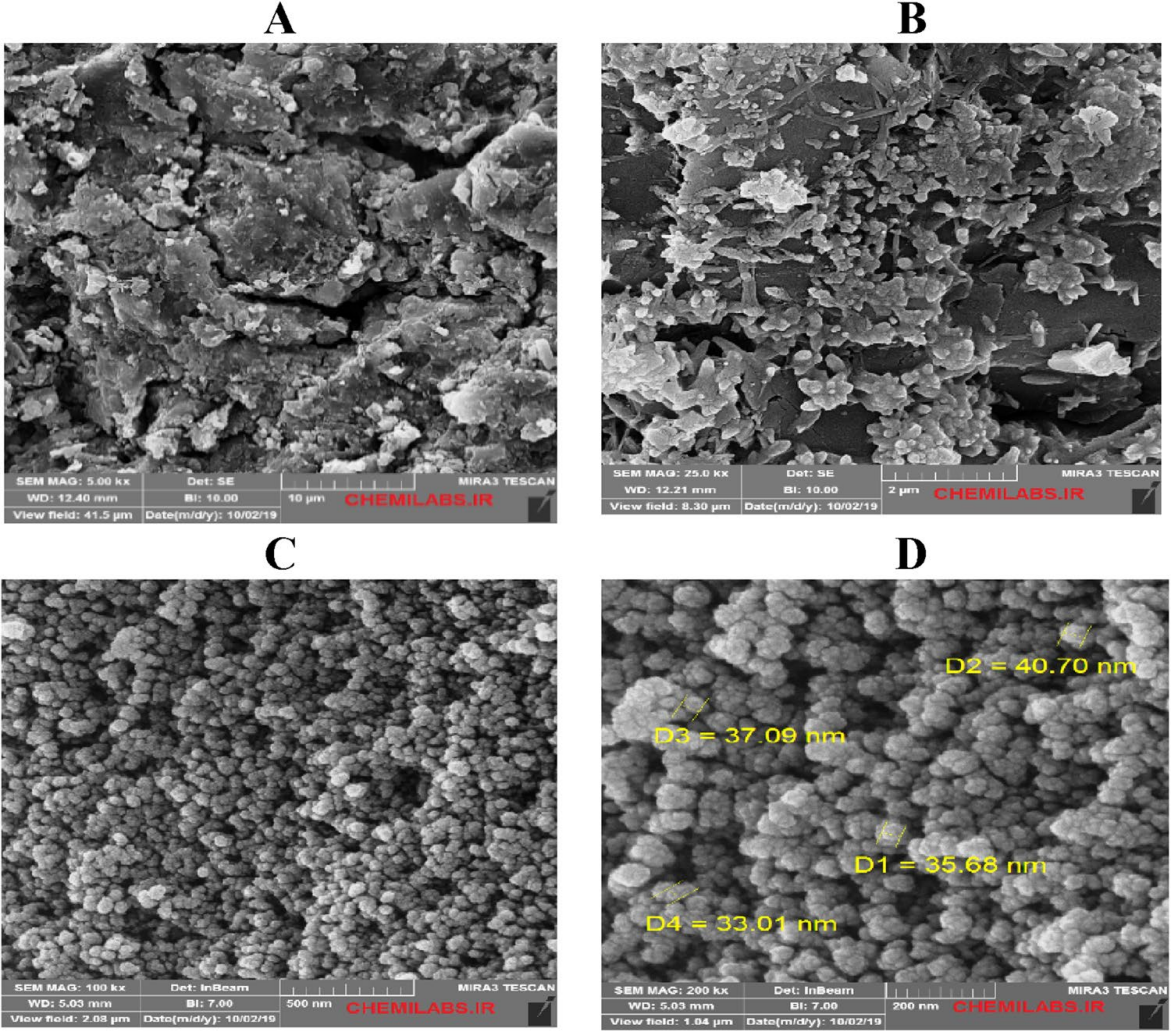
FESEM images analysis of (**A**) a clean sandstone rock (water wet), (**B**) a sandstone rock aged in oil (oil-wet) (**C,D**), and an oil-wet sandstone rock aged in silica nanofluid.

As shown in Figs. 8 and 9, the ZP values from brines/sandstone rock particle samples were negative. Sandstone surface has some active sites for potential determining ions, such as Ca^2+^, Mg^2+^, and SO_4_^2-^ to attach and alter the rock-brine interface charge. Therefore, when the salinity of injection brines changes, the ionic compositions for binding sites and their reaction with OH^−^ and H^+^ ions in solution also change, resulting in ZP values that can differ ^49^. The OLSW solution prompted a higher negative surface charge due to ions adsorption on the rock particles' surface. As the salinity of LSW composition had an optimum salinity, ZP values changed negatively. As a result, by reducing salinity and ionic strength of brine (OLSHW > OLSSW), the ZP gives more negative values (Fig. 10). Also, in brine with larger TDS or lower conductivity, the electrical double layer (EDL) becomes smaller. Consequently, ions accumulate on the EDL and prevent the release of active ions^5^.

The ZP behavior was in agreement with previous experimental studies, which recommend more reduction in salinity of IW changed rock wettability to a less oil-wetting state^5,62–69^. As a result, reduction of ionic strength due to decreasing injected water salinity and utilizing the optimum concentration of IW with minimum scaling are the two effective points that change the rock surface's wettability toward water-wet, resulting in the oil recovery can increase from the oil reservoir^5^. According to Figs. 8, the lower ZP of OLSSW than OLSHW resulted in the lower electrostatic attraction of rock-brine and oil-brine interfaces, hence less oil-wet sandstone surface and incremental oil recovery in the presence of OLSSW (Fig. 15A, C). Finally, the highest ZP value was achieved at pH  4.5, and the surface charge of surface rock was decreased at higher pH. According to the literature, the effect of divalent ions could contest with H^+^ and OH^-^ ions for attraction on the rock surface and create a surface complex^62,70^. A reduction in pH value (pH  4.5–6.5) can increase the surface concentration of CaSO_4_^-^ complexes, leading to a negative surface charge on the surface rock. When the pH value of the solution increases, this process can be reversed. In this condition, the surface concentration of CaSO_4_^−^ decreases and CaOH^0^ complexes increase, but CO_3_Ca^+^ and CO_3_Mg^+^ complexes do not change and cannot be significantly influenced by pH.

Consequently, the net surface charges shift from minor negative to high positive, resulting in an enhancement in ZP value versus a rise in pH ^62^. When divalent cations remove from brine composition, therefore the salinity and TDS of LSSW compared with LSHW are reduced. As a result, a reduction in the ZP value toward additional negative values can obtain in this condition. The main reason was the expansion of the double layer at more down salinity and increasing negative surface charge owing to the alteration in the value of surface complexes^70^. As can be seen in Figs. 8 and 9, the surface charge of rock powder in the presence of NPs was higher than OLSW. In addition, the concentration of Nanosilica in OLSW and the ZP values increased. When silica NPs added to LSW due to their negative surface charge of silica NPs, they can create a nanofluid with a more negative surface charge. As a result, the ZP values of LSHW/LSSW-based nanofluid compositions are increased compared with LSHW/LSSW compositions.

### FESEM analysis

Figure 10 illustrates the FESEM images of surface sandstone rock before and after injection of the silica NPs. Also, Fig. 10A,B show the sandstone plate's surface before aging (water wet) and after surface aging by oil (oil-wet), respectively. After changing the sandstone plate's wettability, as mentioned before, the sandstone surface was aged in nanofluid for 24 h. Figure 10C,D show the FESEM images for the surface of sandstone rock after aging in the nanofluid. The FESEM images show NPs well settle and adsorption on rock surfaces like a coating layer. As a result, silica NPs with negative charges can adsorb on the quartz surface. Also, the tendency and amount of wettability change should be examined to study the effect of rock-OLSW and NPs associated with OLSW interaction on ultimate oil recovery. According to results obtained from FESEM tests in Figs. 10 and 12, the main reasons for increasing oil recovery during silica NPs injection was the adsorption of NPs on rock surface that led to wettability alteration toward more water-wetness. Therefore, the impact of dispersed NPs in OLSW compositions (LSHW and LSSW) on wettability alteration was studied by measuring contact angle values^71^.

Figure 12 shows the contact angle of OLSW compositions (OLSSW, OLSHW, and OLSSW/OLSHW-based nanofluids)/crude oil/sandstone rock system. The contact angle measurements displayed the wettability alteration toward more water-wetness in the presence NPs. As a result, FESEM and contact angle findings confirmed ZP results, which meant that dispersed NPs in LSW were able to make sandstone surface more negative wettability changes from oil-wet toward water-wet.

### Rock-oil-nanofluid system and wettability alteration

**Figure 11:**
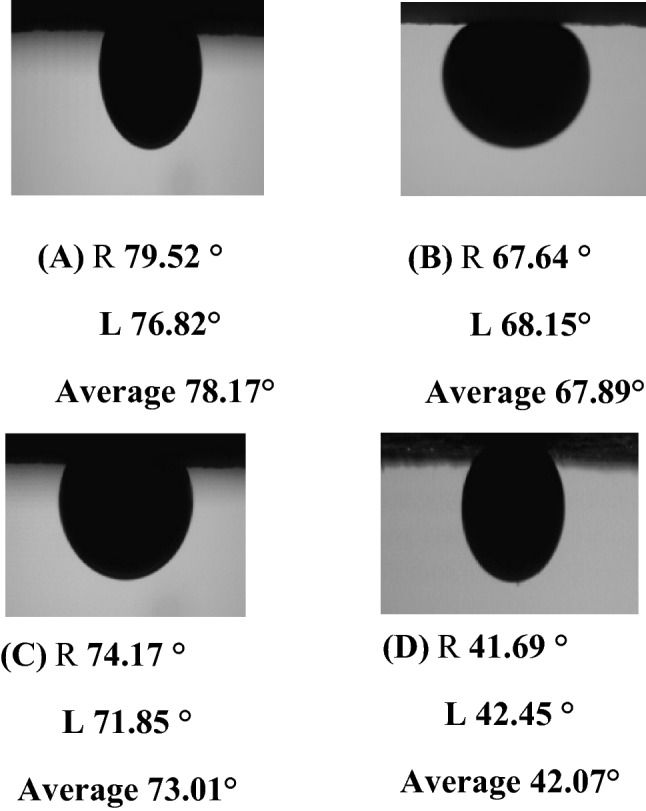
(**A**) Contact angle values of the oil-wetting thin section in the presence of LSHW, (**B**) LSHWN, (**C**) LSSW, (**D**) LSSWN.

Figure 11 shows the crude oil-rock measurements in the presence of OLSSW, OLSHW, and prepared OLSW-based nanofluid solutions. The contact angle was measured based on the wall and oil droplet's right and left sides (R and L), and then the average values were calculated for each sample. Also, the outcomes of contact angle measurements were applied to estimate the achievement of LSW and OLSW-based nanofluid suspensions in wettability alteration to water-wet of surface rock and the effect of silica NPs in this method. As a result, the wettability alteration index (WAI) as a dimensionless number was presented and estimated for each composition as follows:

4 $$ WAI = \frac{{\theta_{0} - \theta_{f} }}{{\,\theta_{0} - \theta_{i} }} $$

**Figure 12:**
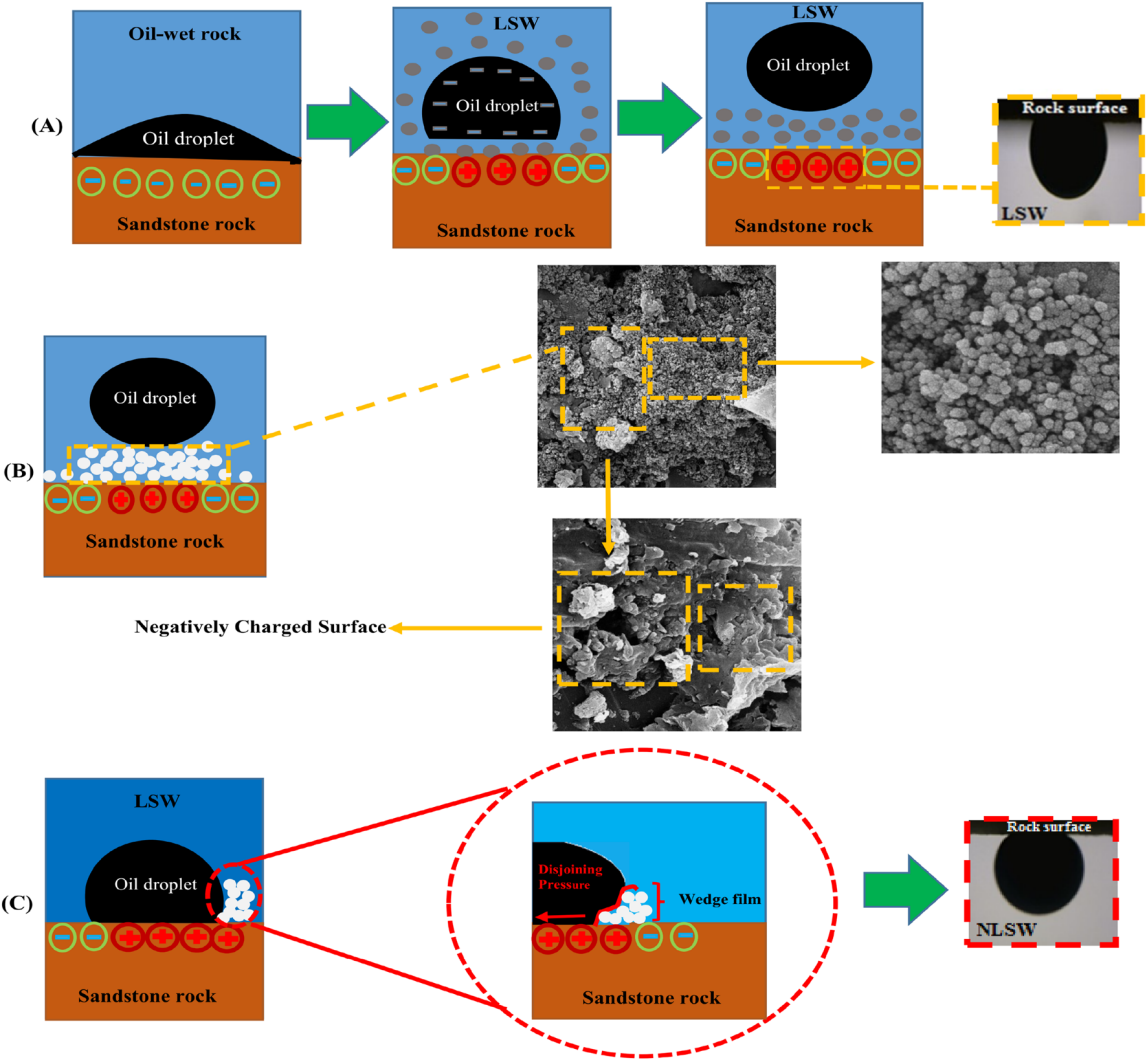
The silica NPs adsorption and surface coating process can be led to wettability alteration of oil-wet toward water-wet conditions. (**A**) Multiple ion exchange (MIE) and EDL attraction between oil droplet and oil-wet sandstone rock surface due to LSW injection, (**B**) wettability alteration toward less oil-wet (more water-wet) due to disjoining pressure utilized by silica NPs in OLSW, and contact angle measurement after one day for LSWN, and (**C**) oil droplet released of rock surface due to surface coating by silica NPs and wettability alteration created by disjoining pressure together with FESEM images obtained from the treated rock surface by nanofluid and contact angle measurement of an oil droplet on the of the thin section in the OLSW.

A WAI near 0 presents that the composition was not efficient in changing the wettability of the sandstone samples, while a WIA near 1 shows complete wettability alteration^30,72^. Table 16 shows the WIA results of different brine compositions. As depicted in Fig. 13A,C, the crude oil-rock contact angle in OLSSW and OLSHW decreased from 122.27° to 78.17° and 107.15° to 73.01°, respectively. The obtained contact angle of LSSW was about 5° lower than LSHW at reservoir temperature conditions (Fig. 11). It might be due to the chemical brine properties that act as a significant impact on the DLE and chemistry of the pore surface, which could govern the interaction of brine-rock and oil-brine interfaces (Fig. 12A). Also, divalent cations of Ca^2+^ and Mg^2+^ decrease ZP value because they tend to aggregate particles (as seen in Tables 11 and 14), shrinking the EDL surrounding the rock surface. Therefore, when divalent cations remove from the LSW solution, the monovalent cations can disperse and increase the space between the interacting surfaces by expanding the external area surrounding the surfaces (diffused layer in EDL) ^69^. In addition, when divalent cations remove from LSSW composition, the salinity and TDS of LSSW compared with LSHW are reduced. Therefore, the reduction in ZP value due to the change of TDS and salinity at oil/LSSW and rock/LSSW systems is adequate. As a result, the attractive force between oil and rock surface decreases compared to LSHW, and wettability alteration can be shifted to the water-wet condition^62^. According to Fig. 11 and Tables 11 and 16, LSSW compared with LSHW can improve the wettability towards strongly water-wet. Also, in the presence of silica NPs in LSHW and LSSW, the oil/rock contact angle reduced to 67.89° and 42.45° (Fig. 11B,D), respectively. As shown in Fig. 12, when the silica NPs inject into the rock-oil–water system, the contact time between the pores walls and nanofluid increases. Then, the NPs in the system form a thin film recognized as wedge films among the rock surface and oil droplets (Fig. 12B). Finally, more oil droplets detached from the rock surface ^71,73^. This film creates excessive pressure on the surface of the oil droplets, named disjoining pressure, which ultimately releases the oil droplets from the pore walls or surface rock (Figs. 12B,C, and 10C,D). This pressure is driven by the electrostatic repulsion between molecules and the nanoparticle's Brownian motion ^73,74^. When salt exists in the non-aqueous solution, it can decrease the repulsive forces between the NPs. More particles can be adsorbed on the rock surface; this work helps create excessive pressure on the surface of oil droplets ^71^. According to results obtained from ZP tests, by removing divalent cation ions in the nano-aqueous solution, more NPs were attached to the surface, and the disjointing pressure increased. As a result, in the presence of nano-silica in LSSW compared with LSHW, the rock wettability altered to strongly water-wet and water-wet conditions (Fig. 11B,D and Table 16). Also, as can be seen in Table 16 that OLSSW based nanofluid compared with OLSHW could improve wettability alteration by about 33.44%. Therefore, it can be concluded there is a close connection between the stability of silica nanofluids and their EOR capacity related to the type and concentration of salt and ion in nano-aqueous solution. Table 17 compares the effects of silica NPs as nanofluids in different brines on wettability alteration in this study with previous works.

**Table 16:** Results of wettability alteration for different LSW compositions with and without silica NPs.

Composition	WAI	Improved wettability alteration
LSHW	0.77	–
LSSW	0.86	–
LSHWN	0.95	23.37
LSSWN	1.34	55.81%

**Table 17:** Comparison between the effect of silica NPs in the LSW solution in this study with previous works.

Technique	Method	Main results	Ref
Dynamic experiment, IFT measurement, and contact angle measurement	Combined nanofluid and LSW flooding	Creating formation damage at high concentration of NPs Final contact angle at 0.05 wt. % of NPs (optimum concentration) = 31^°^ Increasing wettability alteration with increasing concentration of NPs in water-wet system	^75^
Viscosity measurement, IFT measurements, and microfluidic experiments	Water/nanofluid injection	Increasing oil recovery due to Wettability alteration from oil-wet to water-wet by the dispersed silica NPS in injected SW Not measured and recorded contact angle	^23^
Static experiments on NPs stability, contact angle measurement, and imbibition test on EOR	Silica nanofluid flooding	Reduction in contact angle and wettability alteration from oil-wet to water-wet by water base silica nanofluid compared to alkaline fluid Final contact angle = more than 40^°^ Higher oil recovery by silica NPs in water-based nanofluid compared with alkaline fluid	^24^
Contact angle measurement and core flooding test	Hybrid LSW/silica nanofluid flooding	Wettability alteration from oil-wet to water-wet by the dispersed silica NPS in injected SW Final contact angle = more than 60^°^ Incremental 6% oil recovery by injected Nps	^71^
Static test on nanoparticle absorption (ultraviolet (UV) absorption) and automated centrifuge system	Hybrid LSW/silica nanofluid injection	Wettability index = 0.35 Wettability alteration from water-wet to more water-wet by the dispersed silica NPS in injected LSW	^76^
ZP measurement, viscosity measurement, contact angle measurement, and core flooding test	Hybrid LSW/silica nanofluid injection (LSW containing potassium ions + silica NPs)	Final contact angle at 0.05 wt. % of NPs (optimum concentration) = 35.3^°^ Increment of 4% oil recovery by injected Nps	^77^
ZP measurement, contact angle measurement, and scanning electron microscopic (SEM) apparatus	NaCl brine or seawater/silica nanofluid injection	Final contact angle (complex oil-wet system) = 160° Final contact angle (complex water-wet system) = 64.5°	^78^
Dynamic core flood test	Hybrid LSW/silica nanofluid flooding	5–10% Increment in oil recovery by Injected Nps Wettability alteration from water-wet to strongly water-wet by silica nanofluid	^79^
ZP measurement, contact angle measurement, Fesem apparatus	Hybrid SW/silica nanofluid flooding	Final Contact angle (optimized SW + 1500 ppm SiO2) = 79° Increasing wettability alteration by dispersed silica NPs in optimized SW in oil-wet system	^26^
ZP measurement, contact angle measurement, and Fesem apparatus	Hybrid SW/silica nanofluid flooding	Final contact angle (optimized SW + 1500 ppm SiO2) = 45° Increasing wettability alteration by dispersed silica NPs in optimized SW in oil-wet system 10% increment of oil recovery by injected Nps	^44^
ZP apparatus, pH measurement, contact angle measurement, Fesem apparatus, and computability test	Hybrid LSW/silica nanofluid injection	In comparison with previous works, the simultaneous investigation of the effects of different ions, pH, brine compositions, and ZP tests on the stability of NPs in LSW compositions and wettability alteration Final contact angle considering all effective parameters = 42.07^°^ Increasing wettability alteration without formation damage due to OLSSW-based nanofluid compared with OLSHW about 33.44%	This work

## Conclusions

The principal conclusions from this study are summarized as follows:

- The results showed that with decreasing salinity, total cations and anions as TDS, and ionic strength, the stability of NPs in brine increased. Increasing salinity and TDS of injection brine were followed by accelerated nanoparticle aggregation and deposition, destabilizing the nanofluids and retendered unstable colloidal suspensions.
- The compatibility tests and simulation results showed that SW#10D with minimum scale deposition had high nanoparticle stability compared with other times diluted seawater as IW. Therefore, a direct relationship was observed between dilution ratio, scale formation, and stability of NPs in water injection.
- According to the results, a portion of silica NPs can be settled, and nanofluids were obtained from larger particles in nano-aqueous solutions due to Mg^+2^ and Ca^+2^ ions in LSHW composition. Therefore, these causes could negatively affect the potential silica NPs to modify the wettability of surface rock and lead to less oil recovery.
- The surface charge and the ZP values were reduced due to the selective adsorption of ions on the nanoparticle surface that could prevent the particle charge. In addition, these results agreed with the particle size distribution (DLS) analysis, showing that the presence of MgCl_2_ and CaCl_2_ salts could negatively affect the stability of silica nanofluid more than KCl, NaCl, Na_2_SO_4_, and NaHCO_3_.
- The particle size distribution (DLS) of nano-suspensions in the presence of MgCl_2_ and CaCl_2_ salts was more significant than other salts. Therefore, the strength of the repulsive forces between silica NPs in the presence of MgCl_2_ and CaCl_2_ salts in LSHW was lower than of KCl, NaCl, Na_2_SO_4_, and NaHCO_3_ salts in LSSW, leading to more instability of silica nanofluid.
- The ZP results at different pH showed that the ZP values of LSSW solutions in the presence of monovalent and SO^2–^_4_ ions were higher than divalent ions in LSHW. Furthermore, the highest surface charge of silica NPs was obtained in optimal salinity and two optimum concentrations with high stability in the presence of rock at pH  4.5. With increasing pH, this amount was decreased in the presence of rock. Also, according to ZP results that the dispersed silica NPs with optimized concentration into the optimized LSW composition at pH  4.5 could improve the surface charge value by up to 15.71 mV compared with the without NPs.
- The ZP measurements showed that by removing divalent cation ions in the nano-aqueous solution, more NPs were attached to the rock surface, and the disjointing pressure increased. As a result, in the presence of Nanosilica in LSSW compared with LSHW, the rock wettability altered to strongly water-wet and water-wet conditions because divalent Mg^+2^ and Ca^+2^ ions could reduce the nanofluid stability.
- FESEM and contact angle results confirmed ZP results, and it was mean that dispersed NPs in OLSW solutions were able to make sandstone surface more negative wettability changes from oil-wet toward water-wet. Also, according to WAI results, the silica NPs with an optimized concentration in the optimized LSHW and LSSW compositions could improve the wettability alteration by up to 23.37% and 55.81% compared with the without NPs.
- According to WAI results (Table 16), the OLSSW compared with OLSHW could improve the wettability alteration by up to 11.69%. Besides, OLSSW-based nanofluid compared with OLSHW could increase wettability alteration toward strongly water-wet by up to 33.44%.

## Data Availability

The datasets used and/or analyzed during the current study available from the corresponding author on reasonable request.

## Abbreviations

Wt.%: Weight percent
$${\mathrm{\alpha }}_{i}$$: Activity product
$${\mathrm{\alpha }}_{Me}$$: Metal product
Ksp: Solubility product constant
γ_I_: Activity coefficient
${\theta }_{0}$: Original wettability (oil-aged slices)
${\theta }_{i}$: Initial wettability (water-aged slices)
${\theta }_{f}$: Final wettability (oil-aged slices after aging in injected solutions)
